# Cold shock proteins: from cellular mechanisms to pathophysiology and disease

**DOI:** 10.1186/s12964-018-0274-6

**Published:** 2018-09-26

**Authors:** Jonathan A. Lindquist, Peter R. Mertens

**Affiliations:** Clinic for Nephrology and Hypertension, Diabetology and Endocrinology, Otto-von-Guericke University Magdeburg, Leipziger Strasse 44, 39120 Magdeburg, Germany

## Abstract

Cold shock proteins are multifunctional RNA/DNA binding proteins, characterized by the presence of one or more cold shock domains. In humans, the best characterized members of this family are denoted Y-box binding proteins, such as Y-box binding protein-1 (YB-1). Biological activities range from the regulation of transcription, splicing and translation, to the orchestration of exosomal RNA content. Indeed, the secretion of YB-1 from cells via exosomes has opened the door to further potent activities. Evidence links a skewed cold shock protein expression pattern with cancer and inflammatory diseases. In this review the evidence for a causative involvement of cold shock proteins in disease development and progression is summarized. Furthermore, the potential application of cold shock proteins for diagnostics and as targets for therapy is elucidated.

## Background

Imagine proteins that are conserved in both structure and function, that can be found in almost all organisms from bacteria to humans (except yeast), and have been detected in almost every cellular compartment. Add to this the ability to regulate not only their own expression, but the expression of a number of disease-associated genes, and to orchestrate multiple cellular processes, including proliferation and differentiation. Who are these jack-of-all-trades? Enter our protagonists, the cold shock proteins.

### Members of the cold shock protein family

**Fig. 1:**
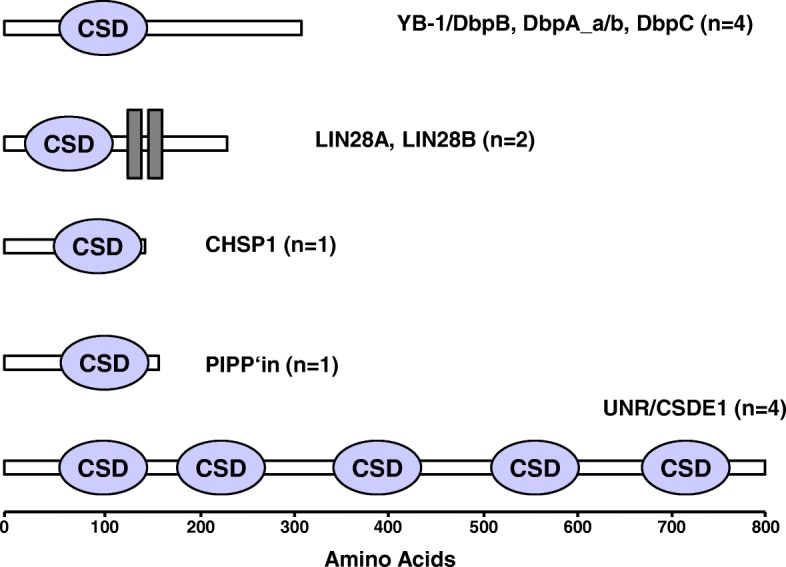
The human cold shock domain proteins. The five groups of human cold shock proteins are presented. The number of proteins in each group is indicated within the brackets. The cold shock domain (CSD) is presented in blue. Lin28 contains two additional zinc finger domains (grey bars). The numbers below indicate the approximate number of amino acids. Structure predictions were performed using the SMART software [215]

Cold shock proteins are among the most evolutionarily conserved proteins [1–3]. Their distinguishing characteristic is the presence of one or more cold shock domains (CSD), which possess nucleic acid binding properties (see Fig. 1 and Table 1). This endows these proteins with pleiotropic functions, such as the regulation of transcription, translation, and splicing [4, 5].

**Table 1:** Nomenclature of the human cold shock domain proteins.

Gene	Gene synonym	Protein	Alternative names
*YBX1*	*MSY1*	**YB-1**	CSDB, DbpB, NSEP1, EF1A
*YBX2*	*MSY2*	**DbpC**	Contrin
*YBX3*	*MSY3/MSY4*	DbpA*	CSDA, ZONAB, oxyR, NF-GMB, YB-2
*CARHSP1*		**CARHSP1**	CSDC1, CRHSP-24, CHSP1
*CSDC2*		**PIPPin**	
*CSDE1*		UNR*	
*LIN28A*		**LIN28A**	CSDD1
*LIN28B*		**LIN28B**	CSDD2

The gene names (italics), common names (bold), as well as commonly used alternative names are presented for each protein. Abbreviations are as follows: Y-box binding protein 1, 2, 3 (*YBX1, YBX2, YBX3*), mouse Y-box protein 1, 2, 3, 4 (MSY1, MSY2, MSY3, MSY4), cold shock domain A, B, C1, C2, D1, D2, E1 (CSDA-CSDE1), calcium-regulated heat stable protein 1 (CARHSP1, CHSP1), calcium regulated heat stable protein 24 kDa (CRHSP-24), abnormal cell lineage protein 28 homolog A, B (LIN28A), DNA binding protein A, B, C (DbpA, DbpB, DbpC), Y-box binding protein 1, 2 (YB-1, YB-2), upstream of N-Ras (UNR), nuclease sensitive element binding protein 1 (NSEP1), enhancer factor I subunit A (EF1A, rat), ZO-1-associated nucleic acid-binding protein (ZONAB), oxidative stress regulatory protein (oxyR), nuclear factor that binds the GM-CSF promoter b (NF-GMB). *Alternatively spliced protein: DbpA has two isoforms, which differ by a single domain of ~ 70 amino acids, whereas the UNR isoforms differ by 31 amino acids

Cold shock proteins were initially identified in bacteria, where a sudden drop in temperature (from 37 °C to 10 °C) induced a 200-fold increase in cold shock protein A (CspA) expression within minutes, which was independent of transcriptional activity [3, 6]. This rapid inducibility is conserved amongst species [7]. A recent study revisited the original observation using genome-wide methods to analyze the global changes occurring in bacteria during the cold shock response [8]. The authors identified RNase R and CspA to be the major players. RNase R appears to be responsible for degrading misfolded RNAs, while CspA melts double-stranded RNAs to enable translation.

In humans, the predominant group of cold shock domain proteins is denoted the Y-box protein family. The prototypic member is Y-box binding protein-1 (YB-1), also known as DNA binding protein B (DbpB), encoded by the gene *YBX1*. Two additional family members exist, DNA binding protein A (DbpA) and C (DbpC), which are encoded by the genes *YBX3* and *YBX2*, respectively.

Whereas *Ybx2* expression is restricted to germ cells [9], *Ybx1* and *Ybx3* are ubiquitously expressed during development. However, following birth the expression of *Ybx3* (DbpA) is down-regulated in most tissues, the exceptions being heart, skeletal muscle, blood vessels, and testis [10, 11]. In humans, two isoforms of DbpA are reported (DbpA_a and DbpA_b), which differ by an alternatively spliced exon that encodes the 69 amino acid long unique domain located adjacent to the CSD [12, 13].

The *Ybx1* knockout mouse is embryonic lethal indicating an important role during development [14]. The *Ybx3* knockout is viable, however the *Ybx1/Ybx3* double knockout shows a more severe developmental phenotype indicating overlapping activities during development [15].

Another developmentally important cold shock protein expressed in humans is Lin28, which was first characterized as a developmental factor in *C. elegans* [16]. However, it was its potential for cellular reprogramming that brought it into the spotlight, as together with Oct3, Sox2, and Nanog, Lin28 is able to revert differentiated cells into their pluripotent state [17]. In addition to the cold shock domain, Lin28A/B are unique in that they also possess two CCHC type zinc fingers, which form a knuckle domain that also participates in nucleic acid binding [18]. Of particular note is the ability of Lin28 to repress let-7 miRNAs, e.g. thereby regulating glucose metabolism [18, 19]. let-7 also targets Lin28 creating a double-negative feedback loop [20]. In addition to miRNAs, Lin28 also binds to mRNAs, participating in a number of ribonucleoprotein complexes, such as P-bodies and stress granules, to regulate translation [21].

A further member of the human cold shock protein family is the calcium-regulated heat-stable protein 1 (CARHSP1); a 24 kDa protein also known as CRHSP-24. Originally identified as a substrate of the calcium/calmodulin-regulated protein phosphatase calcineurin [22], CARHSP1 is a paralog of the brain-specific cold shock protein PIPPin [23]. CARHSP1 binds to and stabilizes tumor necrosis factor (TNF) mRNA within P-bodies and exosomes [24].

PIPPin expression is restricted to brain, where it binds mRNA to regulate translation [25–29]. PIPPin is found with ribonucleoprotein complexes, where it interacts with other RNA binding proteins, e.g. hnRNP A1, hnRNP K, and YB-1 [30].

The final member of this family is denoted *upstream of N-RAS* (*UNR*) [31, 32]. This gene was initially identified as a regulator of N-Ras expression [33–36]. Later it was discovered that *UNR* encodes a protein possessing 5 cold shock domains, which undergoes alternative splicing (see Fig. 1) [37–39]; the gene was then renamed *cold shock domain containing E1* (*CSDE1*). Like the other cold shock proteins, UNR/CSDE1 binds single-stranded DNA or RNA [37, 40, 41]. UNR works together with the polypyrimidine-tract-binding protein (PTB) to regulate translation and mRNA stability [42, 43]. The generation of *Unr* knockout mice demonstrated that, like *Ybx1*, it is essential for mouse development. Further characterization demonstrated that Unr maintains the pluripotent state of embryonic stem cells [44, 45].

As mentioned above, cold shock proteins are components of ribonucleoprotein complexes. Two recent studies using proximity biotinylation to map components of the stress granules identified YB-1, DbpA, CSDE1, and Lin28B [46, 47]. Additionally, CHSP1 (a paralog of PIPPin) was shown to colocalize with G3BP1, an initiator of stress granule formation in human cells [24, 48, 49].

## Cold shock proteins: Thinking in regulatory feedforward and feedback loops

**Fig. 2:**
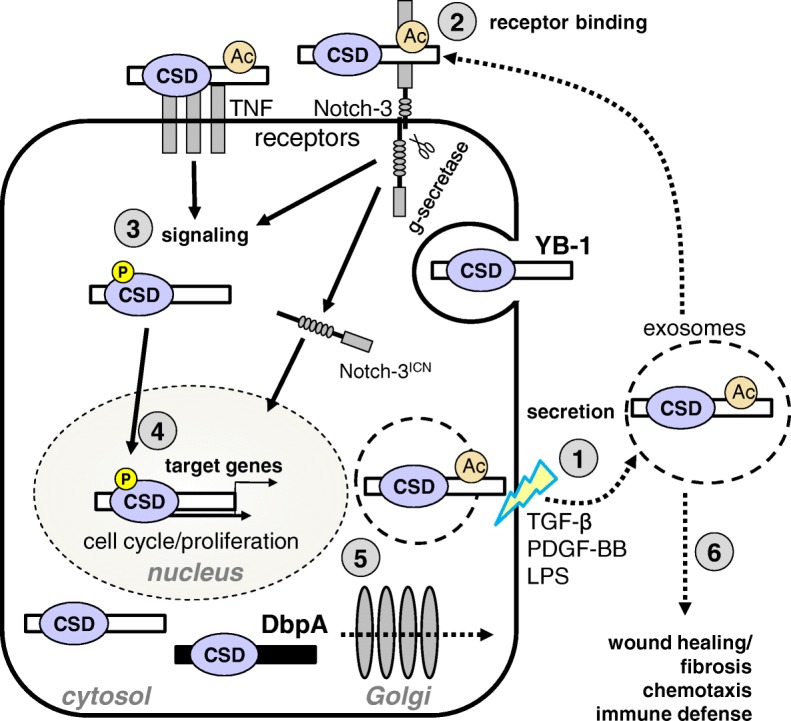
Potential amplification loop for YB-1 in inflammation. (1) Extracellular stimuli (e.g. TGF-β, PDGF-B, LPS) activate cells and induce YB-1 secretion. (2) YB-1 binds to specific membrane associated receptors on the cell surface inducing intracellular signaling cascades that result in kinase activation. YB-1 can also be endocytosed. (3) Activated kinases (e.g. Akt/PKB, ERK, JAK2, RSK) phosphorylate cytoplasmic YB-1 (indicated by the yellow circle), inducing its nuclear translocation. (4) In the nucleus, YB-1 activates the transcription of target genes, as well as induces its own expression and that of DbpA. Cold shock proteins are rapidly induced in response to cell stress, due in part to the existence of preformed complexes of cold shock proteins with their cognate mRNA. (5) Activated cells may also secrete YB-1, which may then act in either an autocrine or paracrine manner. Activated cells may also secrete DbpA via the Golgi. (6) Extracellular YB-1 has mitogenic activity that promotes wound healing/fibrosis. YB-1 also contributes to the recruitment of immune cells to the site of inflammation; directly via its chemoattractant activity or indirectly via the products of its target genes, e.g. CCL5/RANTES. Extracellular activities for DbpA await elucidation. Abbreviations: acetylation (Ac); cold shock domain (CSD); DNA binding protein A (DbpA); lipopolysaccharide (LPS); phosphorylation (P); platelet-derived growth factor B homodimer (PDGF-BB); transforming growth factor beta (TGF-β); tumor necrosis factor (TNF); Y-box binding protein 1 (YB-1)

Cells undergo stress in many ways, e.g. via interferon release in response to viral infection, the presence of lipopolysaccharide produced by bacteria, or profibrotic factors released by immune cells during inflammation. The binding of these factors to their cell surface receptors activates kinases, which phosphorylate the cold shock proteins; here we use YB-1 as an example (see Fig. 2). Upon activation, these RNA/DNA chaperones release specific mRNA, thereby enabling a rapid translational response and translocate to the nucleus to regulate gene expression. In many ways this is similar to the unfolded protein response (UPR) observed for heat shock proteins [50]. The uptake of YB-1 by cells, which is secreted as an RNA:protein complex [51, 52], uniquely positions this cold shock protein to participate in cellular reprogramming by modulating the expression of numerous target genes. Many of these target genes are themselves known to regulate various aspects of disease both intra- and extracellularly (see Table 2) and can induce cold shock protein expression, e.g. PDGF-B and TGF-β. This is envisioned to result in a feedforward amplification loop that prolongs inflammation, promotes cell proliferation and immune cell infiltration, as well as drives fibrosis, analogous to an avalanche [5, 53]. Indeed this scenario has recently been documented, supporting our goal for targeted intervention. How this circuit is terminated is unclear, however the development of cold shock protein targeting “neutralizing” antibodies presents one possibility [54]. Other potential mechanisms include the inducible proteolytic degradation of YB-1 protein, microRNA-mediated inhibition of YB-1 expression, and the induction of protein tyrosine phosphatase activity to counteract the kinase-mediated phosphorylation/activation that induces nuclear protein translocation [55–58].

**Table 2:** Genes regulated by cold shock proteins in disease

Protein	Disease	Target Cell	Mode of Action	Target Gene	Ref.
YB-1	Sepsis	neutrophils, macrophages	N.D.	Toll-like receptor 4 (TLR4) CXCL-1	[123]
	T-cell activation Autoimmunity Inflammation	T-helper cells	binding and stabilization of mRNA	Interleukin 2 (IL-2)	[129, 204]
	Allergic asthma	activated eosinophils	stabilization and up-regulation of mRNA transcripts	GM-CSF	[140]
		embryonic lung fibroblasts	suppression of gene transcription	GM-CSF	[205]
	Chronic liver disease	activated hepatic stellate cells	induction of expression; antagonizes TGFβ signaling	Smad7	[153]
	Chronic liver disease	rat hepatoma cells (FAO)	suppression of gene transcription	Mrp2	[206]
	Kidney transplant rejection	primary monocytes	activation of gene transcription	RANTES/CCL5	[126]
	Kidney transplant rejection	differentiated macrophages	suppression of gene transcription	RANTES/CCL5	[126]
	Neointimal hyperplasia Atherosclerosis	vascular smooth muscle cells	activation of gene transcription	RANTES/CCL5	[127]
	Endometriosis	peritoneal macrophages	activation of gene transcription and recruitment of inflammatory cells	RANTES/CCL5*	[207, 208]
	Chronic kidney disease Interstial kidney disease	proximal tubular cells	control of translation	TGFβ	[132, 209]
	Mesangioproliferative glomerulonephritis	endothelial cells	gene transcription	PDGF-B	[111]
	Mesangioproliferative glomerulonephritis	renal cells	gene transcription, secretion	PDGF-B	[138]
	Tubulointerstial nephritis	renal cells, macrophages	gene transcription, secretion, differentiation, phagocytosis	RANTES/CCL5 MCP-1/CCL2 IL-10	[124, 203]
	Dysregulated angiogenesis		repression of VEGF promotor	VEGF	[210]
	Calcineurin inhibitor mediated kidney fibrosis	mesangial cells	binding and stabilization of mRNA	Collagen	[136]
	Anti-Thy1.1 nephritis	mesangial cells	gene transcription, secretion	Notch-3	[54]
	Type II diabetes	skeletal muscle	gene transcription, signal pathways	PTP1B	[55]
	T-ALL	T cell	Cell cycle	Cdk6	[181]
CHSP1	Inflammation Sepsis	macrophages	enhancement of mRNA stability	TNF	[24]
DbpA	Dysregulated angiogenesis	fibroblasts	repression of VEGF promoter	VEGF	[130, 210]
	Hepatocellular carcinoma	hepatocytes			[211–214]
	Mesangioproliferative glomerulonephritis	renal cells	gene transcription, secretion	DbpA	[13]

For the studied cold shock domain proteins, the disease, target cell, mode of action, and target genes are listed, together with the relevant citation. In sepsis, the mode of action has not been determined (N.D.). Modified from Lindquist et al. [4].

## Cold shock proteins function in the cellular response to stress

Components of stress granules and P-bodies have been implicated in the cellular stress response [59, 60]. Under ‘normal’ conditions, stress granules form when translation initiation is stalled. The RNA binding proteins G3BP1 or TIA-1 are key components of stress granule formation, as they possess the ability for self-association. Over-expression of either protein has been shown to induce stress granule formation even in the absence of stress [49, 61, 62]. Using mRNA as a scaffold, these proteins form homo- or hetero-oligomeric ribonucleoprotein complexes; self-assembly is mediated by intrinsically disordered regions (IDRs) within the RNA binding protein(s); also referred to as low complexity regions [63–68]. Several genetic mutations associated with neurodegenerative diseases have been identified that influence the self-assembly of RNA binding proteins (e.g. *transactive response DNA-binding protein (TDP-43)* and *fused in sarcoma/translocated in liposarcoma (FUS/TLS)*). Both are known to form prion-like protein aggregates; an activity attributed to their low complexity regions [67, 68]. The more we learn about the molecular mechanisms underlying protein aggragation diseases, the greater the number of RNA binding proteins identified [69–71]. The mutations identified within these diseaseassociated proteins typically favor cytoplasmic localization, facilitate protein aggregation, or prevent granulophagy; the clearance of stress granules by autophagosomes [49, 66, 70, 72]. Recently, the expansion of intronic GGGGCC repeats within C9ORF72 was identified as a common cause of ALS/FTD [73]. C9ORF72 interacts with endosomes and is required for normal vesicle trafficking, therefore the loss of C9ORF72 observed with G4-repeat expansion may affect granulophagy. Alternatively, the G4-repeats of C9ORF72 have been proposed to inhibit the neuroprotective effects mediated by tiRNAs binding to the cold shock domain of YB-1 [74].

As a known component of stress granules, YB-1 also possesses the ability for self-assembly [75]. YB-1 has been shown to form amyloid-like fibrils, an activity attributed to its C-terminal domain, which is composed of alternating regions of positive or negatively charged amino acids that form a zipper-like structure as well as contributes to its RNA binding activity [76–81]. Interestingly, the oligomerization of YB-1 is induced by a select set of RNAs [79]. In the context of neurodegeneration, YB-1 and G3BP1 have been shown to compete with TDP-43 and FUS for mRNA binding and thereby induce the release of prion-like protein aggregates that have formed [82]. To complicate matters further, in human sarcoma YB-1 activates *G3BP1* mRNA thereby controlling both the expression levels of G3BP1 and the subsequent nucleation of stress granule formation [83]. Indeed cold shock is one trigger of stress granule assembly in mammals [84]. Stress granules have been implicated in the pathophysiology for a number of neurodegenerative diseases, including Alzheimer’s, amyotrophic lateral sclerosis (ALS), fronto-temporal dementia (FTD), spinocerebellar ataxia (SCA), and Huntington’s disease [49, 71, 85]. Here we propose possible mechanisms where cold shock proteins may play a critical role in the pathophysiology of these diseases. When granulophagy is defective either due to an inability to degrade protein aggregates or to system overload, i.e. when the rate of production exceeds degradation, stress granules that would normally undergo autophagy become lysosomes [64]. The autophagic pathway intersects with both the classical and the unconventional pathways of protein secretion [86, 87]. YB-1 is secreted via a non-classical pathway involving ATP-binding cassette transporters and microvesicles, as well as post-translational modification of two C-terminal lysine residues (K301/K304) [88, 89]. Non-canonical K27-linked ubiquitination of YB-1 was shown to be required for its interaction with tumor susceptibility gene 101 (TSG101), a component of multivesicular bodies (MVBs) [90]. Fusion of MVB with the plasma membrane is required for the release of exosomes [91]. Since YB-1 is a component of exosomes, required for the sorting of mRNAs [51, 52, 92–94], it remains to be determined whether stress granule clearance coincides with the pathway of exosome formation and YB-1 secretion. If these pathways are indeed one in the same, does this apply to both cytoplasmic and nuclear stress granules? Should this hypothesis hold true for YB-1, it will be of interest to see whether it also applies to other cold shock proteins, such as DbpA or CSDE1, that have already been identified as components of both stress granules and exosomes [95, 96].

## Cold shock proteins in disease

The decisive data for a causal relationship between cold shock proteins and disease comes from cancer. The role of YB-1 as an oncoprotein was secured when it was demonstrated that 100% of YB-1 transgenic mice over-expressing the protein developed invasive tumors [97]. YB-1 and DbpA expression is upregulated in cancer and nuclear localization indicates a poor prognosis [57, 98]. In the nucleus, cold shock proteins bind to single- and double-stranded DNA and serve as transcriptional regulators. In tumors, nuclear YB-1 correlates with enhanced expression of the multidrug resistance protein 1 (MDR1) [99–105]. Cells in which YB-1 expression has been ablated using small inhibitory RNA fail to proliferate and were recently shown to prevent tumor growth by disrupting angiogenesis [106].

YB-1 can also be secreted [88]. Acetylation and ubiquitination of YB-1 have both been shown to play roles in regulating secretion as well as intracellular stability [58, 90, 107, 108]. YB-1 can be proteolytically cleaved and extracellular YB-1, and/or fragments thereof, is found in the serum of patients, binds to cell surface receptors, and exerts extracellular activities, e.g. enhancing proliferation and induces migration of immune cells [56, 89, 109–114].

Serum YB-1 levels are increased in cancer patients and the occurrence of extracellular YB-1 or its fragments may serve as a useful marker for cancer, as ~ 80% of patients tested positive for the YB-1/p18 fragment, whereas inflammatory diseases did not correlate with positive results [98, 112–115].

Lin28 reactivation is also found in a number of cancers, where Lin28 appears to contribute to the formation of cancer stem cells [18]. The role of Lin28 in cancer has been extensively reviewed elsewhere [116]. Similar to Lin28, *Unr* also regulates the differentiation state of cells [44]. Due to its ability to regulate the expression of several proto-oncogenes, *UNR* has also been investigated in cancer [117–119]. In prostate cancer, a novel regulatory activity of *HEPSIN* on *UNR* was identified [120, 121]. *UNR* expression levels have also been demonstrated as a prognostic biomarker for survival in pancreatic ductal adenocarcinoma [122].

For inflammatory and fibrotic diseases, the data for the role of cold shock proteins appears more associative. The initial data came from animal studies on *Ybx1* heterozygous mice, which express only half the amount of YB-1 compared to wild type. The induction of disease in experimental models such as sterile sepsis or unilateral ureter obstruction identified non-redundant roles for YB-1 in the development of inflammation and fibrosis [123, 124]. These activities are mediated in part by YB-1-dependent gene regulation of pro-inflammatory factors (PDGF-B, VEGF, IL-2, GM-CSF, EGF, TGF-β, CCL2, CCL5, and CXCR4) [111, 125–134] as well as fibrosis-related genes (*MMP2*, *Col1a1*, and *Col2a1*) (see Table 2) [135–137]. In mesangioproliferative glomerulonephritis, cold shock protein expression is clearly induced; an effect mediated by PDGF-B, and regulates mesangial cell proliferation [13, 138]. In atherosclerosis, YB-1 contributes to neointima formation by modulating CCL5 expression [126, 127, 139]. In asthma, YB-1 promotes eosinophil survival by stabilizing granulocyte macrophage-colony-stimulating factor mRNA [140, 141]. Successful approaches to ameliorate diseases by targeting YB-1 activities have been demonstrated [124, 142–146].

## From molecules to intervention strategies: Rationale for cold shock protein targeting

We propose that cold shock proteins represent verifiable targets for therapeutic intervention and envision strategies aimed at targeting cold shock proteins directly or targeting cold shock protein-dependent mechanisms. This goal is supported by the following observations that link the prototypic cold shock protein YB-1 with other key molecule activities. For the latter, intervention strategies have already proven to be successful.

1. YB-1 regulates NF-κB activation. In the absence of YB-1, NF-κB activation is defective [147, 148].
2. YB-1 regulates IL-2 production. CD28 co-stimulation is required for T cell activation and the induction of autocrine IL-2 production. CD28 signals stabilize IL-2 mRNA. YB-1 is one of the essential RNA binding proteins that mediate this activity [129].
3. YB-1 interacts with p53. Nuclear YB-1 regulates p53 function by inhibiting its ability to induce apoptosis, however it does not influence p53’s regulation of cell cycle [149–151].
4. YB-1 and TGF-β counter-regulate one another. It was recently demonstrated that TGF-β induces miR-216a, which suppresses YB-1 expression. YB-1 suppresses Tsc22, which serves as an enhancer for *Col1a2* expression [152]. Additionally, we have shown that YB-1 mediates the anti-fibrotic effect of interferon-gamma, directly competes for Smad3 binding to p300/CBP [153].

Molecular pathways are not per se pathological, but rather part of regulatory networks. A prolonged or permanent dysregulation results in diseases, especially those of an inflammatory or malignant nature. Developing targeted therapies requires insight into the molecular pathways of underlying diseases, as pivotal cell decisions are dependent on the “activation” of key molecules. Examples of such molecules are provided in the following.

### NF-κB; diseases: Cancer, inflammatory, and autoimmune

Nuclear factor binding near the kappa-light-chain gene in B cells (NF-κB) are a family of inducible transcription factors that control inflammatory gene expression [154–157]. In many cancers, NF-κB is constitutively active and localized to the nucleus. Therefore many anti-tumor therapies seek to block NF-κB activity as a means to inhibit tumor growth or to sensitize tumor cells to conventional therapies, such as chemotherapy. The extensive involvement of NF-κB in inflammation and disease has also established it as a therapeutic target. Indeed, many common synthetic (e.g., aspirin, ibuprofen, glucocorticoids) and traditional medicines (e.g., green tea, curcumin) target the NF-κB pathway. To date, over 800 compounds have been shown to inhibit NF-κB signaling (such as anatabine, disulfiram, dithiocarbamates, olmesartan). Many natural products (including anti-oxidants) that have been promoted to have anti-cancer and anti-inflammatory activity have also been shown to inhibit NF-κB.

### IL-2; diseases: Autoimmune and organ transplantation; cancer, viral infection, and vaccination

Interleukin-2 (IL-2) is essential for lymphocyte survival, differentiation, and proliferation [158–161]. Therefore, many immunosuppressive drugs (such as corticosteroids, cyclosporine A, and tacrolimus) used to treat autoimmune diseases or suppress graft rejection work by inhibiting the production of IL-2 by antigen-activated T cells. Sirolimus blocks intracellular IL-2R signaling, thereby preventing the clonal expansion of activated T cells. The extracellular effects of IL-2 are abrogated by monoclonal antibody application. The use of antibody induction after kidney transplantation has increased to 60% in the past decade and roughly one half of the induction agent used is anti-interleukin-2 receptor alpha antibody (IL-2RA, i.e. basiliximab or daclizumab). In combination with calcineurin inhibitors, IL-2RAs have been shown to reduce the incidence of acute rejection without increasing risks of infections or malignancies in kidney transplantation.

Recombinant IL-2 has been approved for the treatment of cancers (malignant melanoma, renal cell cancer) and has been tested in clinical trials for the treatment of chronic viral infections, and as an adjuvant for vaccines.

### p53; disease: Cancer

Tumor protein p53 (p53) is a tumor suppressor and the most frequently mutated gene in human cancers [162–165]. People who possess only one functional copy of the p53 gene have a higher incidence of tumor development. The p53 gene can also be damaged by chemical mutagenesis or radiation, as well as p53 protein inactivated by viruses (e.g. human papillomavirus). p53 itself does not bind to DNA, but rather exerts its influence via its complex interactions with transcription factors and regulators. p53 mutants are associated with changes in chromatin structure, leading to genetic instability and alterations in cell cycle regulation as well as cellular metabolism. Mutant p53 has been shown to act downstream of the TNF receptor to prolong and enhance NF-κB activation thereby driving tumor-promoting inflammation and enhancing chemokine secretion. The p53 pathway inhibitors nutlin and PRIMA-1 reactivate p53 function, enhancing its anti-proliferative activity and thereby sensitizing cancer cells to apoptosis [166].

### TGF-β; diseases: Organ fibrosis, cancer, immune suppression

Transforming growth factor-β (TGF-β) promotes fibroblast proliferation, differentiation, and survival. In addition to inducing cytokine secretion, TGF-β upregulates the synthesis of collagens and extracellular matrix, making it a therapeutic target in fibrotic diseases [167]. TGF-β also induces the epithelial-mesenchymal xtransition (EMT); an important step in tumor progression, thus making it a target for anti-cancer therapy [168]. Strategies to target TGF-β include neutralizing monoclonal antibodies targeting TGF-β, monoclonal antibodies targeting the integrin α_v_β_6_ are aimed at preventing the activation of latent TGF-β, and small molecules targeting TGF-β receptor activity. Additionally, some commonly used drugs, e.g. the kinase inhibitor imatinib mesylate, appear to also block TGF-β activities and abrogate fibrotic responses [169, 170]. However, inhibiting TGF-β can also have unwanted effects, such as enhanced immune cell activation (due to the loss of TGF-β-mediated inhibition), hindering implantation during pregnancy, and impaired wound healing (within a normal response to injury).

## Outlook

### Diagnostics and therapy with interventions targeting cold shock proteins

Cold shock protein expression is a suitable biomarker for diverse disease activities [112–114]. The presence of extracellular cold shock proteins, and/or fragments thereof, may serve diagnostic purposes. Beyond their diagnostic potential, we envision that therapeutic interventions targeting cold shock proteins may reduce disease burden, as YB-1 is expected to target pathways distinct from those targeted by current therapies. Therefore, we anticipate at least in some cases synergistic activity with existing therapies.

At present cold shock protein research is on the verge of entering clinical trials in different fields, especially for advanced cancer disease (ongoing trials adopt a vaccination strategy against YB-1 epitope in HER2-negative stage III-IV breast cancer or an oncolytic virotherapy in bladder cancer). In experimental disease models intervention strategies targeting YB-1 reduced inflammation and organ fibrosis [124, 142, 143, 171]. HSc025 was identified in a natural products screen for compounds that suppressed collagen gene expression, i.e. fibrosis [172]. HSc025 promotes nuclear translocation of YB-1, which acts as a suppressor of the gene *COL1A2* (*collagen type I alpha 2*) thereby reducing fibrosis [137, 142, 153, 171, 173–175].

Another compound is the natural product fisetin (3,7,3′,4′-tetrahydroxyflavone); a polyphenolic compound found in plants, also called a flavonoid, that demonstrated anti-cancer as well as anti-inflamatory activity [176, 177]. Fisetin blocks the Akt-mediated phosphorylation of Ser^102^ within the CSD [144, 178]. However, an inhibition of p70S6K, a member of the ribosomal S6 kinase (RSK) family, has also been reported [179]. Molecular modeling proposed that fisetin binds to the CSD of YB-1; if such binding prevents YB-1 from being phosphorylated then this proposal would unify these reports, as both kinases phosphorylate Ser^102^ [144, 180, 181]. Regardless of the mechanism of action, fisetin prevents the nuclear translocation of YB-1 by preventing phosphorylation of the CSD.

## Developing topics in the cold shock protein field

Pro-inflammatory factors, like TNF, activate NF-κB, which induces miR-155 expression. Increased miR-155 suppresses CARHSP1, which stabilizes TNF mRNA; thus, this negative feedback loop relieves chronic inflammation and was shown to play a protective role during atherosclerosis [182].

The modulation of tumor necrosis factor receptor signaling by extracellular cold shock proteins is relevant to a number of diseases, including preeclampsia, diabetic nephropathy, systemic lupus erythematosus, liver fibrosis, and infectious diseases where TNF plays a central role in disease pathology [183]. Additionally, TNF promotes expansion of JAK2V617F positive cells in myeloproliferative neoplasms [184]. Extracellular cold shock proteins are also topics of interest, as is their potential role in fetal-maternal communication during implantation.

Receptor Notch-3 is a developmental receptor that plays an important role in stem cell maintenance as well as in cell differentiation. Known roles include the development thymocytes as well as hepatocellular carcinoma. Strong expression is also found in the placenta and uterus suggesting an important role in pregnancy. Extracellular YB-1 serves as a noncanonical ligand for receptor Notch-3 and therefore its ability to modulate receptor Notch-3 signaling is of relevance [88, 89]. Progranulin has recently been demonstrated as a Notch ligand [185] and therefore YB-1/progranulin may also modulate Notch signaling, which may be of relevance in a number of diseases, e.g. diabetic nephropathy, systemic lupus erythematosus, liver fibrosis, and infectious disease.

The participation of extracellular cold shock proteins in inter-organ communication is another important emerging idea (i.e. endocrine activity). Liver-kidney interactions have recently been described for nonalcoholic fatty liver disease (NAFLD) [186]. Here, the liver is an important source of pro-inflammatory cytokines, which modulate inflammation and renal injury [187, 188]. Chronic kidney disease induces intestinal dysbiosis, which contributes to systemic inflammation (via the production of uremic toxins) thereby promoting NAFLD. Inflammation also drives renal fibrosis, which further reduces kidney function, in so doing enhances the levels of uremic toxins within the blood, creating a self-perpetuating multiorgan disease [189]. Several pro-inflammatory cytokines as well as bacterial toxins, e.g. lipopolysaccharide, induce cold shock protein secretion, which binds to TNF receptors and receptor Notch-3 [89]. Therefore we believe that extracellular cold shock proteins are intimately involved in this cycle.

Finally, evidence is emerging that cold shock proteins may regulate the formation of protein aggregates in neurodegenerative diseases [82]. The role of exosomes in the spreading of neurodegenerative and prion diseases is well documented [190–192]. However, it remains to be determined whether stress granules do indeed serve as precursors for exosomes and if so, to what extent they contribute to the spread of neurodegenerative diseases versus the detoxification of cells by removing protein aggregates or perhaps both. Additionally, it remains to be shown whether the targeting of cold shock proteins in this context might be of therapeutic benefit.

Since many components of stress granules and P-bodies are also targets of autoantibodies, the question remains as to whether this pathway contributes to the generation of autoantibodies against YB-1 [193–196]. Certainly the RNA:protein complexes described as “beads on a string” possess the essential elements (i.e. multiple repeating epitopes) required for the successful activation of B-cells [80, 197].

## Post-translational modifications of cold shock proteins

The number of post-translational modifications identified within cold shock proteins is continually growing [198]. A recent paper described O-GlcNAcylation of YB-1; a post-translational modification linking nutrient and stress sensing to transcriptional and translational regulation [199, 200]. This novel modification was shown to contribute to the oncogenic potential of YB-1 in hepatocellular carcinoma (HCC) and appears to exert its activity within the nucleus, since it also requires phosphorylation of Ser102 within the CSD. O-GlcNAcylation is mediated by the enzyme O-GlcNAc transferase (OGT), which is known to promote liver cancer as well as a number of diseases, such as diabetes and neurodegeneration [200, 201]. Since O-GlcNAcylation of NF-κB potentiates its acetylation [202], it will be interesting to see whether a similar effect is also found for the acetylation of YB-1. As you see from this example, there is still much work to be done in linking a particular post-translational modification to specific protein activities. To extrapolate this idea further, it remains to be seen whether there are cell-specific modifications or activities of the cold shock proteins and whether these apply to particular compartments within the cell (e.g. nucleus, mitochondria, exosomes, etc.). Here, it is anticipated that CRISPR/Cas technology will help in creating and characterizing cell lines with specific point mutations targeting a particular modified amino acid. However, there is still much work to be done in identifying and characterizing cell-specific activities of the cold shock proteins. Our recent study demonstrating cell-specific activities of YB-1 in monocytes and macrophages is likely merely the tip of the iceberg [203]. There are still numerous organs, cell types, and cell subsets (e.g. Th1 versus Th2 cells) awaiting characterization. Therefore strategies aimed at deleting *Ybx1* in specific tissues and/or cell types must consider possible developmental effects when characterizing the phenotypes of such cells. Add to this the presence of cold shock proteins within exosomes and thus their extracellular activities and we have a long road ahead to fully understand the complex behavior and activities of these fascinating proteins in both health and disease. Here, the application of high-throughput *omics* technologies will be essential to keep track of the changes going on within such cells on both the transcriptional as well as translational levels.

## Abbreviations

Ac: Acetylation
CARHSP1: Calcium-regulated heat-stable protein 1
CBP: CREB-binding protein
CCL2: Chemokine (C-C motif) ligand 2
CCL5: Chemokine (C-C motif) ligand 5
CRHSP-24: Calcium-regulated heat-stable protein of 24 kDa
CSD: Cold shock domain
CspA: Cold shock protein A
CXCR4: C-X-C motif chemokine receptor 4
DbpA: DNA binding protein A
DbpB: DNA binding protein B
DbpC: DNA binding protein C
EGF: Epidermal growth factor
EMT: Epithelial-mesenchymal transition
FUS/TLS: Fused in sarcoma/translocated in liposarcoma
GM-CSF: Granulocyte-macrophage colony-stimulating factor
HER2: Human epidermal growth factor receptor 2
hnRNP: Heterogeneous nuclear ribonucleoprotein
IL-2: Interleukin-2
IL-2RA: Interleukin-2 receptor alpha
JAK: Janus kinase
LPS: Lipopolysaccharide
MDR1: Multidrug resistance protein 1
mRNA: Messenger RNA
NAFLD: Nonalcoholic fatty liver disease
NF-κB: Nuclear factor binding near the kappa-light-chain gene in B cells
PDGF-B: Platelet-derived growth factor subunit B
TDP-43: transactive response DNA-binding protein
TGF-β: Transforming growth factor beta
TNF: Tumor necrosis factor
UNR: Upstream of N-Ras
UPR: Unfolded protein response
VEGF: Vascular endothelial growth factor
YB-1: Y-box binding protein-1
